# HOXC8 regulates self-renewal, differentiation and transformation of breast cancer stem cells

**DOI:** 10.1186/s12943-017-0605-z

**Published:** 2017-02-16

**Authors:** Mansi Shah, Ryan Cardenas, Belinda Wang, Jenny Persson, Nigel P. Mongan, Anna Grabowska, Cinzia Allegrucci

**Affiliations:** 1 SVMS, University of Nottingham, Sutton Bonington Campus, Loughborough, LE12 5RD UK; 2 Department of Pharmacology, Weill Cornell Medicine, 1300 York Ave., New York, NY 10065 USA; 3 Department of Translational Medicine, Lund University, Malmö, 205 02 Sweden; 4 Department of Molecular Biology, Umeå University, 901 87 Umeå, Sweden; 5 Cancer Biology, Division of Cancer and Stem Cells, School of Medicine, University of Nottingham, QMC, Nottingham, NG7 2UH UK

**Keywords:** Breast cancer, Cancer stem cells, Self-renewal, Differentiation, Homeobox genes, HOXC8

## Abstract

**Background:**

Homeobox genes are master regulators of cell fate during embryonic development and their expression is altered in cancer. By regulating the balance between cell proliferation and differentiation, they maintain homeostasis of normal tissues. Here, we screened the expression of homeobox genes in mammary stem cells to establish their role in stem cells transformation in breast cancer.

**Methods:**

Using a Homeobox Genes PCR array, we screened 83 homeobox genes in normal cancer breast stem/progenitor cells isolated by flow cytometry. The candidate gene *HOXC8* epigenetic regulation was studied by DNA methylation and miRNA expression analyses. Self-renewal and differentiation of HOXC8-overexpressing or knockdown cells were assessed by flow cytometry and mammosphere, 3D matrigel and soft agar assays. Clinical relevance of in vitro findings were validated by bioinformatics analysis of patient datasets from TCGA and METABRIC studies.

**Results:**

In this study we demonstrate altered expression of homeobox genes in breast cancer stem/progenitor cells. *HOXC8* was consistently downregulated in stem/progenitor cells of all breast molecular subtypes, thus representing an interesting tumour suppressor candidate. We show that downregulated expression of *HOXC8* is associated with DNA methylation at the gene promoter and expression of *miR196* family members. Functional studies demonstrated that HOXC8 gain of function induces a decrease in the CD44^+^/CD24^-/low^ cancer stem cell population and proportion of chemoresistant cells, with a concomitant increase in CD24^+^ differentiated cells. Increased HOXC8 levels also decrease the ability of cancer cells to form mammospheres and to grow in anchorage-independent conditions. Furthermore, loss of HOXC8 in non-tumorigenic mammary epithelial cells expands the cancer stem/progenitor cells pool, increases stem cell self-renewal, prevents differentiation induced by retinoic acid and induces a transformed phenotype.

**Conclusions:**

Taken together, our study points to an important role of homeobox genes in breast cancer stem/progenitor cell function and establishes HOXC8 as a suppressor of stemness and transformation in the mammary gland lineage.

**Electronic supplementary material:**

The online version of this article (doi:10.1186/s12943-017-0605-z) contains supplementary material, which is available to authorized users.

## Background

Cancer stem/progenitor cell cells (CSC) are considered to play an important role in breast cancer complexity as they retain the fundamental features of normal stem cells, being able to self-renew and differentiate into the different cell types that comprise the heterogeneous tumour mass [1, 2]. Breast CSC have a degree of cellular plasticity and it is unclear whether they originate from transformation of normal mammary stem cells (MaSC) or by reprogramming of differentiated cells to a malignant phenotype [1].

Genes that are required for tissue development and normal stem cell plasticity may contribute to the molecular blueprint of breast CSC and tumorigenesis. Indeed, it is well established that many molecular pathways involved in normal development are altered during cancer progression [1]. Amongst the developmental pathways, homeobox genes function as “master” gene regulators. They are characterised by a homeodomain that binds specific DNA elements and encode transcription factors involved in the regulation of cell growth and differentiation, thus establishing and maintaining cell identity and fate during development [3]. Given their role, it has been postulated that a balanced expression of these genes is critical for stem cell function, especially for self-renewal, differentiation and quiescence [4]. Within homeobox genes, the HOX gene family is important in early tissue differentiation. For instance, the HOXA gene cluster is silenced in pluripotent human embryonic stem cells and epigenetically reprogrammed and expressed when cells are induced to differentiate [5]. They also regulate neural and hematopoietic stem cells function [6, 7].

Homeobox genes are expressed during mammary gland development and they appear to be regulated both by hormones and extracellular matrix remodelling [8]. Examples include *Hoxc6* and *Msx2* being down-regulated and up-regulated by estrogen and progesterone, respectively. *Msx2* is also regulated by the interaction between epithelial and stromal cells in the gland [8]. Several homeobox genes are involved in proliferation and differentiation of mammary stem/progenitor cells. For instance *Prrx1, Six1*, *Lbx1*, *Sox9*, *Msx, Zeb1*, *Dlx4* have been shown to regulate epithelial-to-mesenchymal transition (EMT) in mammary cells, thus promoting stem cell-like characteristics [9–15].

Aberrant expression of homeobox genes has been reported in several malignancies [4]. Mis-expression of homeobox genes can lead to abnormal differentiation and proliferation, leading to a change in cell identity or homeotic transformation, therefore playing an important role in carcinogenesis [16]. In cancer, homeobox genes function as “tumour modulators” as their deregulation normally involve either up-regulation of genes expressed in undifferentiated cells or down-regulation of genes expressed in differentiated tissue, thus acting either as oncogenes or tumour suppressor genes [17]. Abnormal expression of homeobox genes leading to loss of differentiation is observed in breast cancer where expression of *Hoxb6*, *Hoxb7*, *Hoxc6*, *Hoxc8*, *Hoxd4*, *Hoxd8*, *Hoxd9*, *Hoxd10, Hoxa5* is lost in mouse mammary tumours and *Hoxa1*, *Hoxd3*, *Hoxd12*, *Msx1*, *Six1* and *Oct3* are instead expressed in cancer, but absent or lowly expressed in the normal differentiated gland [8]. This deregulated gene expression has been shown to be involved in neoplastic transformation by regulating cell cycle, apoptosis, angiogenesis, metastasis and cell adhesion [8]. Epigenetic mechanisms, such as DNA methylation, histone modification and silencing by non-coding RNA are involved in the regulation of homeobox gene expression [4]. Moreover, epigenetic silencing of these genes is an early event in breast carcinogenesis [18–20] and DNA methylation of homeobox genes is associated with specific breast cancer cell types and cancer molecular subtypes [21–23].

In this study, we screened the expression of homeobox genes in breast CSC and demonstrate that a large number of genes are differentially expressed in immortalised CSC compared to normal MaSC, in agreement with evidence suggesting an involvement of developmentally-regulated genes in early stages of cancer transformation. We also reported expression of homebox genes uniquely altered in different breast cancer subtypes, which could represent novel CSC biomarkers for patient stratification. Of the genes commonly altered across different breast cancer molecular subtypes, *HOXC8* was chosen as a putative novel tumour suppressor gene. We show that *HOXC8* downregulation in breast CSC is associated with epigenetic silencing. Downregulation of *HOXC8* in breast cancer was also found by meta-analysis of breast cancer data from large cohort studies. Gain of function of *HOXC8* reduced CSC self-renewal and the ability of cancer cells to grow in anchorage-independent conditions. Conversely, loss of function of *HOXC8* in mammary normal cells induced CSC proliferation and colony formation. In addition, reduced *HOXC8* expression impaired cell differentiation and response to retinoic acid. Taken together, our study shows that homeobox genes represent novel biomarkers of breast CSC and that *HOXC8* functions as a novel tumour suppressor gene by regulating breast CSC proliferation and differentiation.

## Methods

### Cells and materials

Human mammary epithelial cells (HMEC) were obtained from Invitrogen and cultured with the proprietary HuMEC Ready Medium. The immortalised human mammary epithelial cell line MCF10A (ATCC) was cultured in HuMEC Ready Medium supplemented with 100 ng/ml cholera toxin. Breast cancer cell lines MCF-7, HCC1954, HCC1428 (all from ATCC), MDA-MB-468, MDA-MB-231, BT549 and Hs578T (all from the NCI-60 cell collection, CRN cell bank, University of Nottingham) were grown in RPMI medium supplemented with 10% foetal calf serum (FCS), 1% Penicillin/Streptomycin (Pen/Strep), 1% L-Glutamine, 1% sodium pyruvate, 1% non-essential amino acids (NEAA). HEK 293 T (ATCC) cells were grown in DMEM medium containing 10% FCS supplemented, 1% Pen/Strep, 1% L-Glutamine, 1% sodium pyruvate, 1% NEAA. Cell lines obtained from ATCC were used within few passages from the original stocks, whereas other cell lines were authenticated by STR profiling (Eurofins Genomics, Germany) using the Promega PowerPlex 21 PCR kit and matched against the ATCC STR database. All cell lines were tested form mycoplasma contamination using the EZ-PCR Mycoplasma Test Kit (Geneflow). All cell culture materials were from Invitrogen and chemicals from Sigma-Aldrich, unless otherwise stated.

### Cell treatments

For chemoresistance assay, cells were incubated with Paclitaxel (10nM), doxorubicin (500 nM) and 5-Fluorouracil (5-FU) (500 μM) for 5 days. Viable cells were trypsinised, collected and the CD44^+^/CD24^-/low^ profile was analysed by flow cytometry.

For differentiation experiments, cells were treated with 1 μM all-*trans*-retinoic acid (ATRA) for 7 days.

### Flow cytometry

Stem/progenitor cell populations were identified and isolated as CD44^+^/CD24^-/low^ by FACS (Beckman Coulter MoFlo XDP sorter). The following antibodies and isotype controls were used: FITC-anti-CD44 (eBioscience 11-0441, 1:100 dilution), PE-anti-CD24 (eBioscience 12-0247, 1:20 dilution), APC-anti-CD44 (eBioscience 17-0441, 1:167 dilution), FITC-anti-CD24 (eBioscience 11-0247, 1:20 dilution), FITC-rat isotype control (eBioscience 11-4031, 1:100 dilution), PE-mouse isotype control (eBioscience 9012-4714, 1:20 dilution), APC-rat isotype control (eBioscience 17-4031, 1:167 dilution), FITC-mouse isotype control (eBioscience 11-4714, 1:20 dilution). Briefly, 5x10^5^ cells were incubated with antibodies in the dark at 4 °C for 1 h. Cells were washed and re-suspended in complete medium for analysis. For cell sorting, 10^6^ cells in 100 μL of complete medium were stained as described above. Cells were then treated with DNase I (Qiagen) in RDD buffer (10 mM Tris-HCL, 2.5 mM MgCl2, 0.5 mM CaCl2, pH7.6) for at least 15 min at room temperature, and passed through a 40 μm mesh (Becton Dickinson LTD) to avoid cell clumping. Data were analysed with the Weasel software.

### Homeobox gene expression array

Total RNA was extracted with the RNeasy Mini Kit (Qiagen) and cDNA transcribed with the RT^2^ First Strand Kit (Qiagen). Expression of homeobox genes was analysed by real-time PCR (qRT-PCR) using the Homeobox (HOX) Genes RT^2^ Profiler PCR Array (Qiagen) on a Roche LightCycler® *480* System. Real-time PCR data analysis was performed by using the RT^2^ Profiler™ PCR Array Data Analysis (Qiagen) and based on the ΔΔC_T_ method with normalization of the raw data to the housekeeping gene *RPLP0* after analysis with the BestKeeper software [24].

### Real-time PCR

Gene expression was analysed by qRT-PCR using TaqMan® chemistry (Applied Biosystems). Data were analysed by the ΔΔC_T_ method with normalization to the housekeeping genes *RPLP0. HOXC8* copy number was measured by qRT-PCR using genomic DNA (extracted with DNeasy Blood &Tissue kit, Qiagen) and LuminoCt® SYBR® Green qPCR ReadyMix. miRNAs were purified from cell pellets using the miRNeasy Mini Kit (Qiagen) and reverse transcribed with miScript II RT kit (Qiagen). qRT-PCR was performed using the LuminoCt® SYBR® Green qPCR ReadyMix. Data were analysed by the ΔΔCT method with normalization to the endogenous control *RNU6B*. For primers and assay used see Additional file 1: Table S1.

### Bisulfite sequencing

Genomic DNA was isolated using DNeasy Tissue kit (Qiagen). Bisulfite conversion (200 ng genomic DNA) was achieved using the EZ DNA methylation kit (Zymo Research) and 3 μl of bisulfite converted DNA was used for PCR reaction using the HotStarTaq masterm mix kit (Qiagen). Primers spanning the CpG island sequence were designed using the Methprimer software (http://www.urogene.org/cgi-bin/methprimer/methprimer.cgi) (Additional file 1: Table S1). Bisulfire converted and purified PCR products were directly sequenced.

### Lentiviral plasmids for HOXC8 overexpression and knockdown

The HOXC8 plasmid was prepared by amplifying *HOXC8* cDNA from HMEC cells using Phusion® high-fidelity DNA polymerase PCR master mix (New England Biolabs) and cloning between EcoRI and SpeI sites of a modified pSIN-EF2-Puro plasmid (pSin-EF2-Nanog-Pur was a gift from James Thomson, Addgene plasmid # 16578). *HOXC8* and scrambled shRNA primers were constructed using previously reported sequences [25]. shRNA harpins were ligated into AgeI/EcoRI-digested pLKO-Tet-On plasmid (Tet-pLKO-puro was a gift from Dmitri Wiederschain, Addgene plasmid # 21915). Plasmids were transformed in Stabl3 cells (Invitrogen) and purified using Qiagen plasmid purification kits (Qiagen). Viral particles were produced by transfecting HEK 293 T cells with shRNA or pSIN plasmids, together with psPAX2 and pMD2.G plamids (gift from Didier Trono, Addgene plasmids # 12260 and 12259) using FuGENE®6 (Promega). Virus-containing supernatant was collected at 72 h post-transfection, filtered through 0.45 μm filter and added of 6 μg/mL hexadimethrine bromide (polybrene) (Sigma-Aldrich). Stable transgenic cells were selected after 72 h using Puromycin (0.75–1 μg/ml). Tet-inducible shRNA was induced by treatment with 2ug/ml doxycycline.

### Western blotting

Nuclear proteins were extracted with NucBuster™ Protein Extraction Kit (Calbiochem). Extracted proteins were loaded into a 12% or 15% Acrylamide gel (20 μg/lane), separated by SDS-PAGE electrophoresis and blotted onto a PVDF membrane. Membranes were blocked with 5% skimmed milk and then probed overnight at 4 °C with a rabbit anti-HOXC8 antibody (1:1,000, Sigma H1791) in the presence of 0.1% Tween 20 and 5% milk. Membranes were stripped and the incubated with a mouse anti-Lamin A/C antibody (1: 2,000, Cell Signalling 4777) overnight at 4 °C. Peroxidase conjugated donkey anti-rabbit and sheep anti-mouse (1:10,000; GE Healthcare NA934 and NA931, respectively) antibodies were incubated for 1 h at RT. ECL prime kit (GE Healthcare) was used to detect chemiluminescence.

### Mammospheres culture

Single cells were plated in ultra-low adherent flasks coated with Poly 2-hydroxyethyl methacrylate (PolyHEMA) (Sigma-Aldrich) at a density of 2x10^4^ cells/mL. Cells were grown in mammosphere medium consisting of Dulbecco’s Modified Eagle Medium: Nutrient Mixture F-12 (DMEM/F12) supplemented with 20 ng/mL epidermal growth factor (EGF, R&D Systems) 20 ng/mL basic fibroblast growth factor (bFGF) (R&D Systems), 5 μg/mL insulin, and 1% pen/strep, B27, 4 μg/mL heparin, and 5 μg/mL hydrocortisone. Cells were incubated at 37 °C and 5% CO_2_ for 7 days to attain first generation mammospheres. Formed mammospheres were collected by gentle centrifugation at 200 × g for 4 min, dissociated enzymatically with trypsin and mechanically by pipetting. Serial passaging to attain consequent generations of mammospheres (up to four) was done by re-plating dissociated mammosphere-derived cells at a density of 5x10^3^ cells/ml and culture for 7–10 days in mammosphere medium. For PKH26 labelled mammospheres, 2x10^6^ cells were stained with 1 μM PKH26 for 5 min, washed and then and plated in mammosphere medium for the first generation. After 7 days, mammospheres were collected, dissociated as described above and sorted to isolate the PKH26 positive^high^ population. Sorted cells were cultured again in mammosphere medium to obtain the second generation.

### Soft agar assay

Cancer cells (1-3x10^4^ cells/6 well) were seeded in 1 ml of 0.5% noble agar in complete RPMI medium overlaying 2 ml 1% agar in the same medium. MCF10A cells were instead seeded in HuMEC medium, as described above. After 2 weeks culture, cell colonies were stained with 0.05% crystal violet in 10% ethanol/PBS for 1 h and colonies ≥ of 100 μm counted under a MZ125 Leica stereomicroscope.

### 3D Matrigel assay

The 3D on-top Matrigel assay was performed according to Lee et al. [26]. Cells (1.5x10^4^) were plated into a 4 well plate covered with 120 μl of growth factor reduced Matrigel (BD Biosciences) and cells allowed to attached for 30 min. Cells were then cultured in 500 μl HuMEC Ready Medium supplemented with 100 ng/ml cholera toxin and 10% Matrigel for up to 7 days, with medium changed every other day.

### Bioinformatics and statistics

For the analysis of the Homeobox gene array, hierarchical cluster analysis was performed by using the software ArrayMining (http://www.arraymining.net/). Venn diagrams were generated with the online tool Venny (http://bioinfogp.cnb.csic.es/tools/venny/index.html).

The analysis of copy number variation in clinical datasets was performed with the software OASIS (OASIS: A Web-based Platform for Exploratory Analysis of Cancer Genome and Transcriptome data, www.oasis-genomics.org). Expression analysis of homeobox genes in TCGA (RNASeq and PAM50) and METABRIC patient datasets was performed using the UCSC Cancer Browser (https://genome-cancer.ucsc.edu/) and the OASIS portal, respectively [27, 28]. For the analysis of *HOXC8* in the TCGA dataset, clinical parameters and next-generation RNA sequencing data (RNAseqv2) were obtained from TCGA ccRCC and normal non-tumour breast tissue sample set [29, 30]. Patients were sub-divided depending on clinic-pathological parameters provided by TCGA. Normalized gene counts (gene counts rescaled according to library size) from each patient were compiled into a tab-delimited file for downstream analysis by RobinA implementation of the Bioconductor-edgeR software [31, 32]. Expression analysis of *HOXC8* in the METABRIC dataset [33, 34] was also obtained from the OASIS portal. Methylation analysis of the TCGA dataset was performed by using the UCSC Cancer Browser and the MethHC browser (http://methhc.mbc.nctu.edu.tw/php/index.php) [35].

All data are expressed as mean ± standard deviation. Comparisons between two samples were done using Student’s *t*-test. One-way or Two-way analysis of variance (ANOVA) analyses followed by Bonferroni’s multiple comparison tests were used for multiple group comparisons. Statistical analyses were performed with GraphPad Prism with significance levels set at *P < 0.05, **P < 0.01, ***P < 0.001.

## Results

### Expression of homeobox genes in breast CSC

In this study we investigated the expression of 83 different homeobox genes involved in morphogenesis, body pattern formation, embryonic development, and cellular differentiation in normal MaSC and breast CSC. We used cell lines representing normal mammary epithelium, immortalised mammary epithelium (IMM) and the breast cancer molecular subtypes luminal (LUM: ER^+^, PR^+^, HER2^−^), HER2-enriched (HER2-E: ER^−^, PR^−^, HER2^+^) and basal/triple negative (TN: ER^−^, PR^−^, HER2^−^). CD44^+^/CD24^-/low^ stem cell/progenitor cells were isolated from normal and immortalised mammary cells (HMEC and MCF10A, respectively), LUM (MCF-7, HCC1428), HER2-E (MDA-MB-468, HCC1954) and TN (BT549, Hs578T and MDA-MB-231) cancer cells. Expression of the majority (77.11%) of the homeobox genes analysed was altered in CSC compared to MaSC (CD44^+^/CD24^-/low^ in HMEC) across different cancer subtypes (≥2-fold change, with genes considered differentially expressed only if either up-regulated or down-regulated in at least 2 cell lines of the same molecular subtype).

**Fig. 1:**
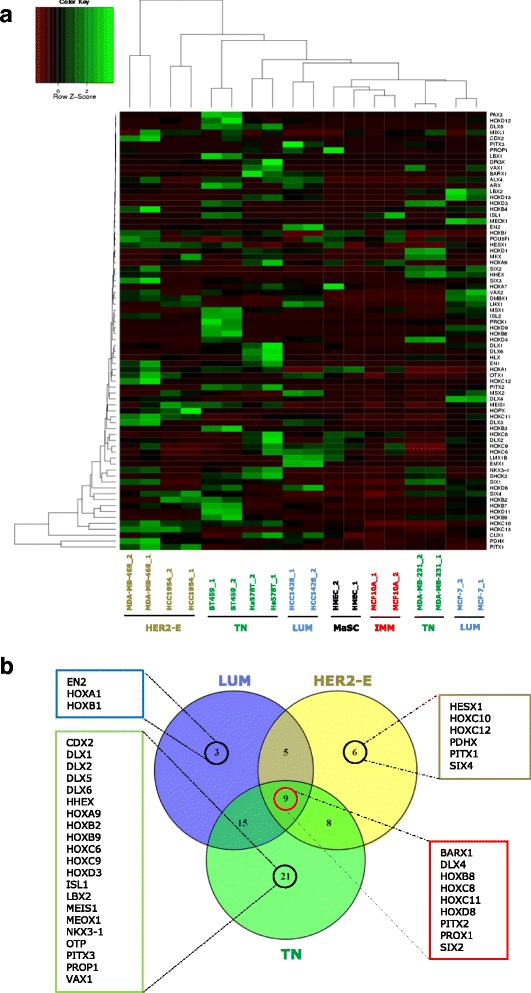
Differential expression of homeobox genes in CSC compared to normal MaSC. **a** Unsupervised hierarchical clustering of 83 homeobox genes based on expression in CSC isolated by CD44^+^/CD24^low/-^ cell sorting of different cell lines representing breast cancer molecular subtypes (LUM, HER2-E, TN), immortalised mammary epithelial cells (IMM) and normal mammary cells. Homeobox gene expression was determined by qRT-PCR using using the Homeobox Genes RT2 Profiler PCR Array (Qiagen) with two independent biological replicates. **b** Venn diagram representing differentially expressed genes unique or in common among CSC of different breast cancer molecular subtypes compared to normal MaSC (CD44^+^/CD24^-/low^ cells from HMEC). Subtype-specific and common differentially expressed genes are indicated in the highlighted boxes

Unsupervised hierarchical cluster analysis showed that HMEC and MCF10A clustered together, showing a similar expression of homeobox genes in non-tumorigenic stem cells. Normal stem cells clustered closer to luminal CSC, whereas HER2-E and TN CSC clustered separately (Fig. 1a). Surprisingly, MDA-MB-231 cells clustered with luminal CSC and not TN CSC, suggesting that this cell line may have fundamental genetic and epigenetic differences compared to the other TN cell lines analysed.

A large number of genes were differentially expressed in immortalised stem cells, suggesting altered expression of homeobox genes is an early event during CSC transformation. The largest number of differentially expressed genes was observed in TN CSC. Differential expression of a large number of genes was consistent across cell lines of the same molecular subtypes, representing 54.8% of the genes in LUM, 41.8% in HER2-E, and 72.2% in TN. A minority of genes changed expression in either only one cell line within molecular subtypes or in different directions (either down- or upregulated). The majority of genes in the immortalised stem cells were downregulated, whereas upregulated genes were predominant in CSC across the different molecular subtypes (Additional file 2: Figure S1A). Of the downregulated genes in the immortal stem cells, the majority were shared by TN CSC, whereas upregulated genes were shared similarly by all CSC subtypes. Interestingly, the TN CSC showed the largest number of uniquely downregulated and upregulated homeobox genes (Additional file 2: Figure S1B, Figure S1C). When considering the cancer subtype-specific homeobox genes, three genes were unique to LUM CSC, 6 to HER2-E CSC, and 21 to TN CSC. Nine genes were instead common to all cancer subtypes (considering at least 2 cell lines) (Fig. 1b). Of these, six genes were either consistently upregulated (*BARX1*, *DLX4*, *HOXB8*, *PITX2*, *SIX2*) or downregulated (*HOXC8*).

**Fig. 2:**
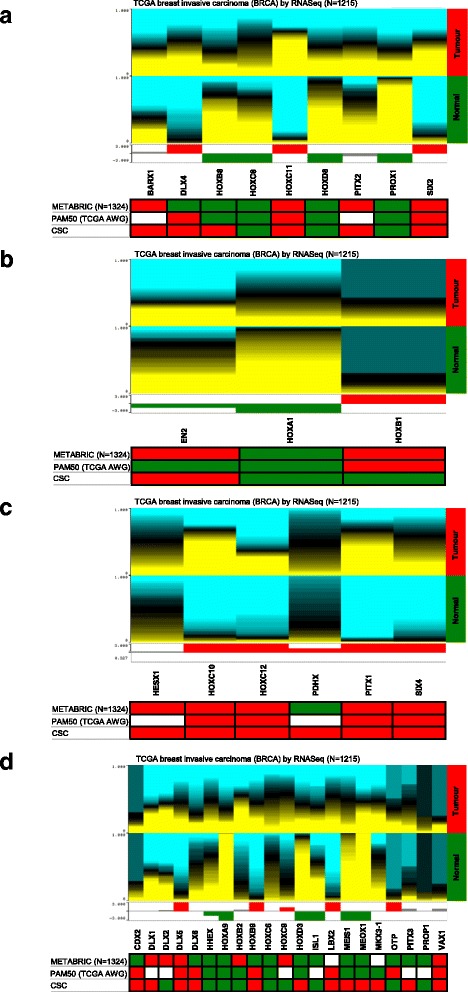
Data heatmaps and histograms showing expression of homeobox genes in the TCGA (RNASeq and PAM50) and METABRIC datasets. Genomic heatmaps compare expression of genes between normal and tumour samples, with *yellow* and *blue* colour intensity indicating high and low expression, respectively. The *red* and *green* colours under the genomic heatmaps indicate the statistics track showing the logarithmic plot of *P*-values for each gene using Student’ *t*-test followed by Bonferroni’s correction. Bars above the *line* indicates that the *red* subgroup (Tumour sample) is greater than the *green* subgroup; a bar below the *line* indicates that the *green* subgroup (Normal sample) is greater than the *red* subgroup. Bars are coloured in *red* or *green* when *P* < 0.05. The same colour code summarises the same information obtained from analysis of the METABRIC and PAM50 datasets (Additional file 3: Figure S2) and the homeobox PCR array (Fig. 1) in the tables below the heatmaps. **a**. Homeobox genes differentially expressed in CSC of all cancer subtypes. **b**. Homeobox genes differentially expressed in LUM CSC. **c**. Homeobox genes differentially expressed in HER2-E CSC. **d**. Homeobox genes differentially expressed in TN CSC

The expression of those genes that were differentially expressed either in all or specific cancer subtypes (Fig. 1b) was further validated by analysis of large cohort patient data from the publically available TCGA and METABRIC datasets [29, 30, 33, 34]. Figure 2 summarises the validation using TCGA RNASeq (all samples or samples classified according to their PAM50/subtype specific signature [30]) and METABRIC data, with genes upregulated in tumours shown as red and downregulated in tumours (upregulated in normal tissue) as green (see also Additional file 3: Figure S2). The majority of homeobox genes altered in breast CSC showed a good overlap of expression with patient tumours (76.3%; 29/38 genes matching at least one dataset), confirming the clinical relevance of our findings. Of the genes that were altered in all cancer subtypes, the expression of five matched all datasets (*HOXC8*, *HOXC11*, *HOXD8*, *PROX1*, *SIX2*), although the overlap was found when considering the expression in the majority of the subtypes as *HOXC11* and *PROX1* were downregulated in TN CSC. *DLX4* matched two datasets (TCGCA and PAM50) and *BARX1* and *PITX1* only the METABRIC dataset. In contrast, no match was found for *HOXB8* expression.

For the luminal-specific genes, *HOXA1* was found downregulated in all datasets, *EN2* upregulated in the METABRIC, and *HOXB1* expression showed no overlap. The genes in the HER2-E subtype corresponded well with patient data, with *HOXC10*, *HOXC12*, *PITX1*, *SIX4* matching all datasets and *HESX1* and *PDHX* the METABRIC and TCGA sets, respectively. Finally, In the TN-specific gene set, *DLX5*, *HHEX*, *HOXA9* expression corresponded to all datasets, whereas *HOXB9*, *HOXC6*, *ISL1*, *LBX2*, *VAX1* only to two datasets (TCGA/PAM50 or TCGA/METABRIC or METABRIC/PAM50). Interestingly, of the genes that overlapped with only one dataset (*CDX2*, *DLX1*, *DLX6*, *HOXB2*, *OTP*, *PROP1*), three matched with their PAM50 subtype (*CDX2*, *DLX6* and *HOXB2*). However, the expression of a number of gene did not correspond to any dataset (*DLX2*, *HOXC9*, *HOXD3*, *MEIS1*, *MEOX1*, *NKX3-1*, *PITX3*), suggesting that the expression of these genes might possibly be specific to the particular cell lines analysed in this study.

### HOXC8 is downregulated in breast CSC and tumour tissues

**Fig. 3:**
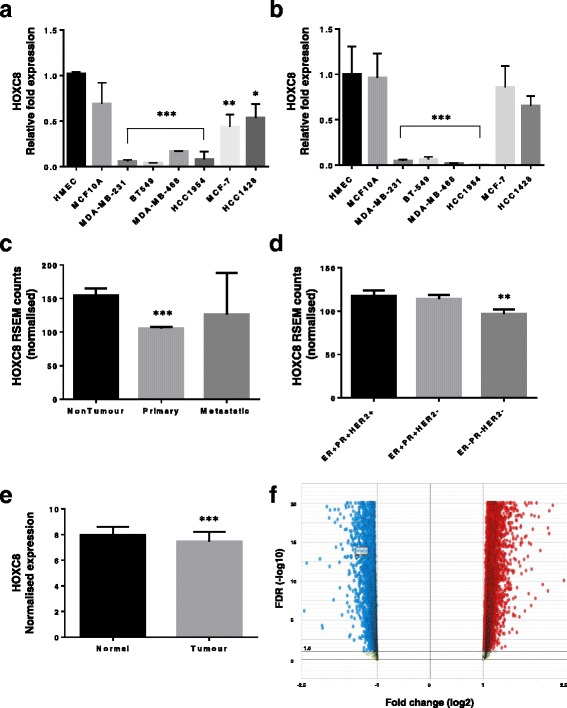
*HOXC8* expression is reduced in breast CSC and breast cancer clinical samples. **a** Expression of *HOXC8* in CSC sorted as CD44^+^/CD24^low/-^ cell population as determined by TaqMan® qRT-PCR. Results are presented as relative fold expression relative to *RPLP0* and HMEC used as calibrator (*n* = 3–4). Relative fold expression levels were analysed by One-way ANOVA followed by Bonferroni’s multiple comparisons test. *P < 0.05, **P < 0.01, ***P < 0.001. **b** Expression of *HOXC8* in unsorted cell lines as determined by TaqMan® qRT-PCR. Results are presented as relative fold expression relative to *RPLP0* and HMEC used as calibrator (*n* = 3–4). Relative fold expression levels were analysed by One-way ANOVA followed by Bonferroni’s multiple comparisons test. ***P < 0.001. **c** Expression of *HOXC8* in patient tumour tissue determined by RNA-Seq in the breast TCGA dataset [29, 30]. Statistical analysis was performed by Bioconductor-edgeR. ***P < 0.001. **d** Expression of *HOXC8* in patient tumour tissue classified according to hormone receptor status as determined by RNA-Seq in the breast TCGA dataset. Statistical analysis was performed by Bioconductor-edgeR. **P < 0.01, ER^+^PR^+^HER2^+^ (*n* = 115), ER^+^PR^+^HER2^−^ (*n* = 408), ER^−^PR^−^HER2^−^ (*n* = 128). **e** Expression of *HOXC8* in patient tumour tissue determined by microarray analysis of the breast cancer METABRIC dataset access through the OASIS software [33, 34]. Statistical analysis was performed by Unpaired Student’s *t*-test. ***P < 0.001. **f** Volcano plot of the METABRIC dataset showing differentially expressed genes in breast cancer patients compared to normal tissue as visualised in the OASIS software. The significant downregulated *HOXC8* expression is highlighted

Our array data showed *HOXC8* downregulation in CSC compared to normal MaSC, suggesting a possible role as tumour suppressor gene that regulates normal function of mammary stem cells. Microarray data was validated by qRT-PCR, with HER2-E and TN CSC showing most significant downregulation (Fig. 3a). This expression profile was mirrored by the unsorted cell lines for HER2-E and TN subtypes, but not for LUM subtype (Fig. 3b). In order to extend our analysis of *HOXC8* to clinical samples, we next analysed the expression of *HOXC8* in the latest TCGA [29, 30] and METABRIC [33, 34] datasets. Analysis of RNA-Seq data of the TCGA patient tumour samples (*n* = 1218) revealed *HOXC8* expression is lower in primary breast tumours compared to non-tumour tissue, whereas no difference was observed in metastatic tumours (Fig. 3c). Significant low expression was also found in TN (ER^−^/PR^−^/HER2^−^) compared with ER^+^ tumours (ER^+^/PR^+^/HER2^+^ or ER^+^/PR^+^/HER2^−^), which is consistent with our findings in CSC (Fig. 3d). The same profile of expression between normal and tumour tissue was also shown by the analysis of the METABRIC dataset (*n* = 1321) (Fig. 3e, f).

### HOXC8 downregulation in breast CSC is associated with epigenetic silencing

**Fig. 4:**
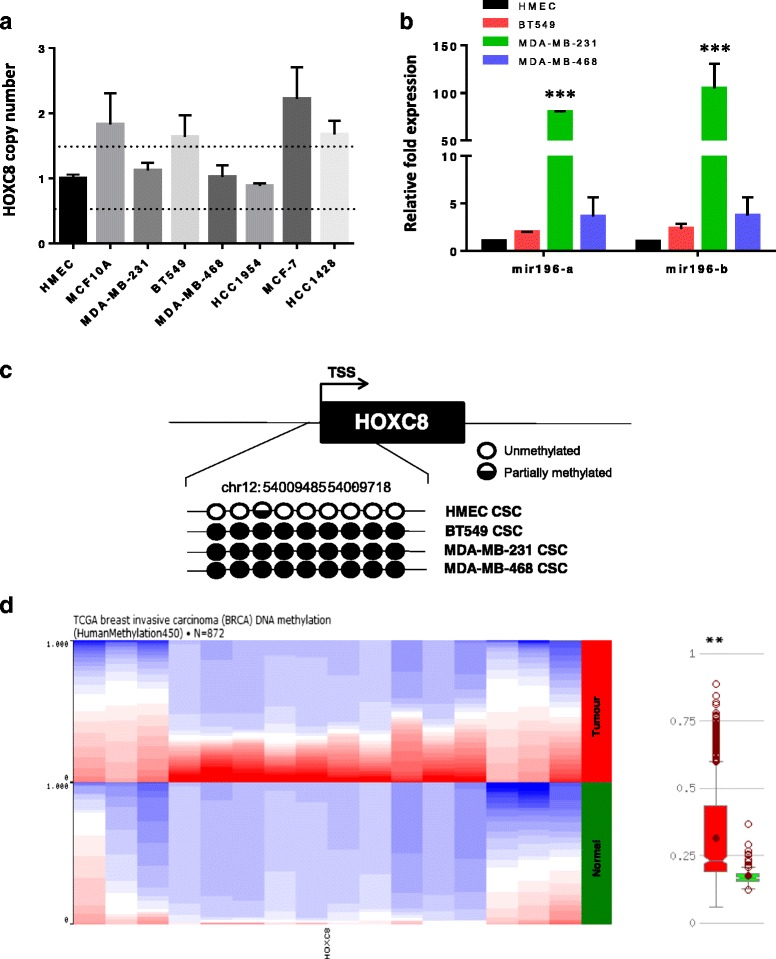
*HOXC8* expression is downregulated in TN and HER2-E CSC by epigenetic silencing. **a** *HOXC8* genomic copy number in CSC sorted as CD44^+^/CD24^low/-^ cell population as determined by SYBR® qRT-PCR. Results are presented as fold difference relative to HMEC used as calibrator (*n* = 4). *Dotted lines* represent the range of normal copy number 1 ± 95% confidence interval. **b** Expression of *miR196-a* and *miR196-b* in CSC sorted as CD44^+^/CD24^low/-^ cell population as determined by SYBR® qRT-PCR. Results are presented as relative fold expression relative to *RNU6B* and HMEC used as calibrator (*n* = 3). Relative fold expression levels were analysed by One-way ANOVA followed by Bonferroni’s multiple comparisons test. ***P < 0.001. **c** DNA methylation profile of *HOXC8* promoter CpG Island. DNA methylation was analysed by direct PCR bisulfite sequencing. *Black circles* indicated methylated CG dinucleotides, *white circles* indicate unmethylated CG dinucleotides, *white/black* semicircles indicate partially methyalted CG dinucleotides. **d** DNA methylation profile of *HOXC8* promoter of TCGA clinical samples profiled by Illumina Infinium HumanMethylation 450 BeadChip array (*n* = 872). *Left panel* shows DNA methylation normalised proportions in normal and tumour samples as visualised in UCSC Cancer Browser (*blue* colour represents lower methylation, *red* colour represents higher methylation). *Right panel* shows DNA methylation levels in tumour samples (*red*) compared to normal (*green*) as visualised by MethHC browser. Statistical analysis was performed by Student’s *t*-test. **P < 0.01

In order to study the regulation of *HOXC8* expression in breast CSC, we next analysed whether downregulation could be due to copy number variation or epigenetic regulation. Analysis of the TCGA [29, 30] and METABRIC [33, 34] breast cancer datasets showed *HOXC8* is rarely deleted in breast cancer and its expression does not correlate with copy number in breast cancer patients (Additional file 4: Figure S3). Similarly, cell lines analysed in this study showed no *HOXC8* deletion when compared to normal mammary epithelial cells (HMEC) or MCF10A cells (Fig. 4a).

Therefore we focused our attention on the epigenetic regulation of *HOXC8*. Expression of *miR196* has been previously linked to regulation of *HOXC8* [36] and therefore we measured the expression of two *miR196* family members in HER2-E and TN CSC, as these showed the most downregulated expression of *HOXC8*. The expression of *miR196-a* and *miR196-b* was significantly increased in CSC isolated from MDA-MB-231, but not in CSC isolated from other cell lines (Fig. 4b). Direct bisulphite sequencing analysis of the CpG island in the *HOXC8* promoter upstream of the transcription start site revealed high levels of DNA methylation in all CSC compared to normal MaSC (Fig. 4c and Additional file 5: Figure S4). A significant increase in DNA methylation at the promoter region of *HOXC8* was also observed in the breast TCGA tumour samples compared to normal tissue (Fig. 4d), thus demonstrating an important role of epigenetic modifications in the regulation of *HOXC8* expression and the clinical relevance of the in vitro findings.

### Gain of function of HOXC8 reduces CSC self-renewal and chemoresistance

**Fig. 5:**
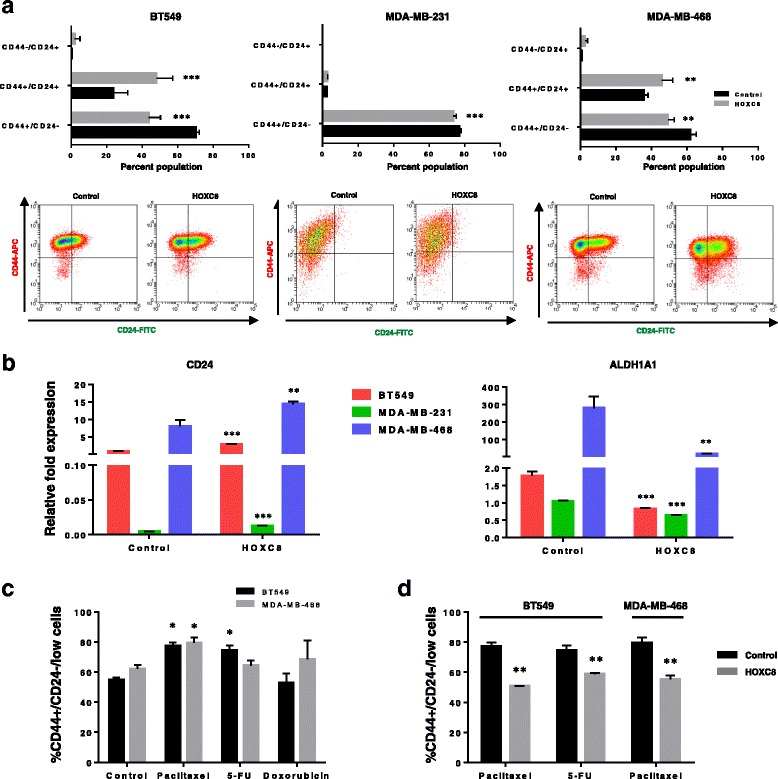
HOXC8 expression negatively regulates CSC and chemoresistance. **a** Representative FACS profiles of cells transduced with pSIN-HOXC8 or pSIN empty control vector and quantification of CSC and non-CSC populations stained with CD44-APC and CD244-FITC. Results were analysed by comparing levels of CD44^+^/CD24^−^, CD44^+^/CD24^+^, CD44^−^/CD24^+^ cells in pSIN-HOXC8 transduced cells compared to control vector for each cell line (*n* = 3). Statistical analysis was performed by Two-way ANOVA followed by Bonferroni’s multiple comparisons test. **P < 0.01, ***P < 0.001. **b** Expression of *CD24* and *ALDH1A1* in Control and HOXC8-overexpressing cells. Gene expression was measured by TaqMan® qRT-PCR. Results are presented as relative fold expression relative to *RPLP0* and control (pSIN vector) used as calibrator (*n* = 3). Relative fold expression levels were analysed by Unpaired Student’s *t*-test. **P < 0.01, ***P < 0.001. **c** Percentage of CD44^+^/CD24^low/-^ cells in cell lines treated with 10nM Paclitaxel, 500 μM 5-FU and 500nM Doxorubicin for 5 days as measured by FACS analysis. Results were analysed by comparing levels of CD44^+^/CD24^low/-^ cells in untreated versus treated cells by One-way ANOVA followed by Bonferroni’s multiple comparisons test for each cell line (*n* = 3). *P < 0.05. **d** Percentage of CD44^+^/CD24^low/-^ cells in cell lines treated with 10nM Paclitaxel, 500 μM 5-FU and 500nM Doxorubicin for 5 days as measured by FACS analysis. Results were analysed by comparing levels of CD44^+^/CD24^low/-^ cells in pSIN-HOXC8 or pSIN empty control vector transduced cells by Unpaired Student’s *t*-test for each cell line (*n* = 3). **P < 0.01

We next assessed the functional role of *HOXC8* in breast CSC by inducing its expression in cancer cell lines. Constitutive overexpression of *HOXC8* in low-expressing cell lines (BT549, MDA-MB-231, MDA-MB-468) resulted in high levels of expression both at the RNA and protein level (Additional file 6: Figure S5). Induced HOXC8 expression caused a significant reduction in the CD44^+^/CD24^-/low^ population (Fig. 5a). The reduction was due to an induction of differentiation reflected by an increased double positive epithelial cell fraction (CD44^+^/CD24^+^) in both BT549 and MDA-MB-468 cells, but not in MDA-MB-231. This effect on CSC fate was further validated by the increased expression of *CD24* and reduced expression of the CSC marker *ALDH1* (Fig. 5b). To explore whether the increased differentiation induced by HOXC8 would affect the resistance of cells to chemotherapy, we treated control BT549 and MDA-MB-468 cells with the standard of care anti-cancer drugs Paclitaxel, Doxorubicin and 5- FU and evaluated the effect of these drugs on the CSC population. Paclitaxel and 5-FU increased the number of CD44^+^/CD24^-/low^ cells after 5 days of treatment, whereas no effect was seen with doxorubicin (Fig. 5c). Consistent with the significant decrease in CSC and increased differentiation in these cell lines, overexpression of *HOXC8* caused a significant reduction in chemoresistant cells (Fig. 5d).

**Fig. 6:**
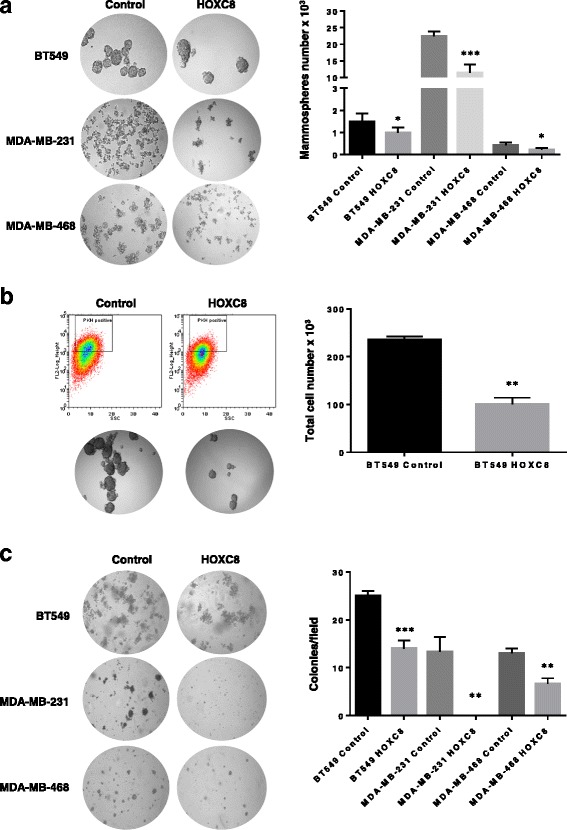
HOXC8 expression reduced CSC self-renewal and anchorage independent cell growth. **a** Mammospheres morphology and number obtained by self-renewal of cell overexpressing HOXC8 or control vector after 4 generations. Mammospheres numbers were compared by Unpaired Student’s *t*-test for each cell line (*n* = 4). *P < 0.05, ***P < 0.001. Only spheres ≥ 100 μM were counted, images were taken at 10X magnification. **b** Second generation mammospheres obtained after labelling with the PKH26 dye and sorting of the PKH26 ^positive/high^ population from first generation mammospheres. Results show the morphology of mammospheres overexpressing HOXC8 or control vector and total mammospheres cell number. Mammospheres numbers were compared by Unaired Student’s *t*-test (*n* = 3). **P < 0.01. Only spheres ≥ 100 μM were counted, images were taken at 10X magnification. **c** Colonies of cells overexpressing HOXC8 or control vector grown in soft agar for 2 weeks. The number of colonies were calculated by counting 10 fields of view. Results were analysed by Unpaired Student’s *t*-test for each cell line (*n* = 3). **P < 0.01, ***P < 0.001. Images were taken at 10X magnification

To further evaluate the effect of *HOXC8* in the regulation of CSC self-renewal, we performed serial passaging of mammospheres at clonal density. The reduced CSC population induced by HOXC8 expression resulted in a significant reduction in mammosphere formation over 4 generations for all the cell lines tested (Fig. 6a), confirming a role of HOXC8 in determining stem cell fate. As the CD44^+^/CD24^−^ population can identify both CSC and early progenitor cells, we also stained HOXC8-expressing cancer cells with the PKH26 dye and studied the ability of HOXC8 to regulated the more primitive and quiescent CSC population which is able to retain the dye upon cell proliferation. Therefore after labelling, the first generation mammospheres were sorted for PKH26^positive/high^ CSC and new mammospheres formed. Overexpression of HOXC8 reduced the ability of quiescent PKH26^positive/high^ CSC to form second generation mammospheres (Fig. 6b). Finally, HOXC8- overexpressing cells displayed a reduced growth in soft agar (Fig. 6c), demonstrating a diminished transformed phenotype.

### Loss of function of HOXC8 increases stem cell self-renewal and transformation

**Fig. 7:**
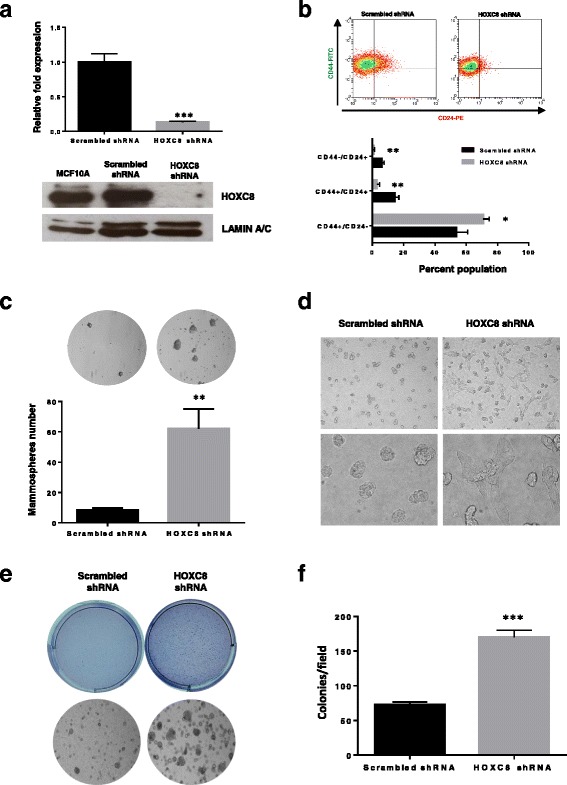
HOXC8 knockdown increase CSC proliferation and transformed phenotype. **a** Knockdown of HOXC8 was induced by lentiviral transduction of MCF10A cells with pLKO-Tet-HOXC8 vector and gene expression measured by TaqMan® qRT-PCR. Results are presented as relative fold expression relative to *RPLP0* and pLKO-Tet-Scrambled vector used as calibrator (*n* = 3). Results were analysed by Unpaired Student’s *t*-test (*n* = 3). ***P < 0.001. Bottom panel shows western blotting of nuclear lysates from cells transduced with pLKO-Tet-HOXC8 vector or pLKO-Tet-Scrambled vector was conducted to detect the expression of HOXC8 (34 kDa) and LAMIN A/C (41–50 kDa) as loading control. **b** Percentage of CD44^+^/CD24^low/-^, CD44^+^/CD24^+^, CD44^−^/CD24^+^ cells after HOXC8 knockdown as measured by FACS analysis. Results were analysed by comparing cell populations levels in HOXC8 shRNA transduced cells compared to Scrambled shRNA (*n* = 3). Statistical analysis was performed by Unpaired Student’s *t*-test. *P < 0.05, **P < 0.01. Representative FACS profiles of cells double stained with CD44-FITC and CD244-PE to analyse the CSC and non-CSC populations are also shown. **c** Mammospheres morphology and number obtained by self-renewal of cell after HOXC8 knockdown compared to scrambled control vector after 3 generations. Mammospheres numbers were compared by Unpaired Student’s *t*-test (*n* = 3). **P < 0.01. Only spheres ≥ 100 μM were counted, images were taken at 10X magnification. **d** Colonies of HOXC8 shRNA and Scrambled shRNA cells grown in 3D matrigel. Top panels represents 10X magnification, bottom panel represents images at 40X magnification. **e** Colonies of cells with HOXC8 knockdown compared to scrambled control grown in soft agar for 2 weeks. Top images show the whole well stained with crystal violet, bottom images show a representative field of view at 10X magnification. **f** The number of soft agar colonies were calculated by counting 10 fields of view. Results were analysed by Unpaired Student’s *t*-test for each cell line (*n* = 3). ***P < 0.001

Because of the proposed role of HOXC8 in the regulation of breast stem cell fate, we next evaluated whether HOXC8 loss of function in non-tumorigenic mammary epithelial cells would be responsible for the acquisition of stemness by differentiated cells. We therefore knocked down *HOXC8* in the immortal but not-tumorigenic MCF10A cells, as stem cells in this cell line expressed similar levels of *HOXC8* to those isolated from normal HMEC cells (Fig. 3a). *HOXC8* shRNA resulted in about 80% knockdown in gene expression and no detectable levels of HOXC8 protein (Fig. 7a). HOXC8 knockdown induced a significant increase in CD44^+^/CD24^-/low^ cells with a concomitant decrease in CD44^+^/CD24^+^ and CD44^−^/CD24^+^ cells, confirming its role in maintaining a differentiated state in the mammary epithelial lineage (Fig. 7b). HOXC8 downregulation also increased the number of mammospheres over several generations (Fig. 7c), indicating that silencing of *HOXC8* is associated with a switch of cell fate and acquisition of stem cell characteristics. Reduced HOXC8 expression also resulted in a loss of cell organisation as cells grown in 3D matrigel appeared to lose their dome-shaped morphology and acquire a mesenchymal/basal phenotype (Fig. 7d). Importantly, HOXC8 knockdown induced a ~ 2.5-fold increase in the number and size of colonies of MCF10A cells grown in anchorage-independent conditions compared to the scrambled control (Fig. 7e, f), indicating an augmented transformed phenotype.

### Expression of HOXC8 is required for breast stem cell differentiation

**Fig. 8:**
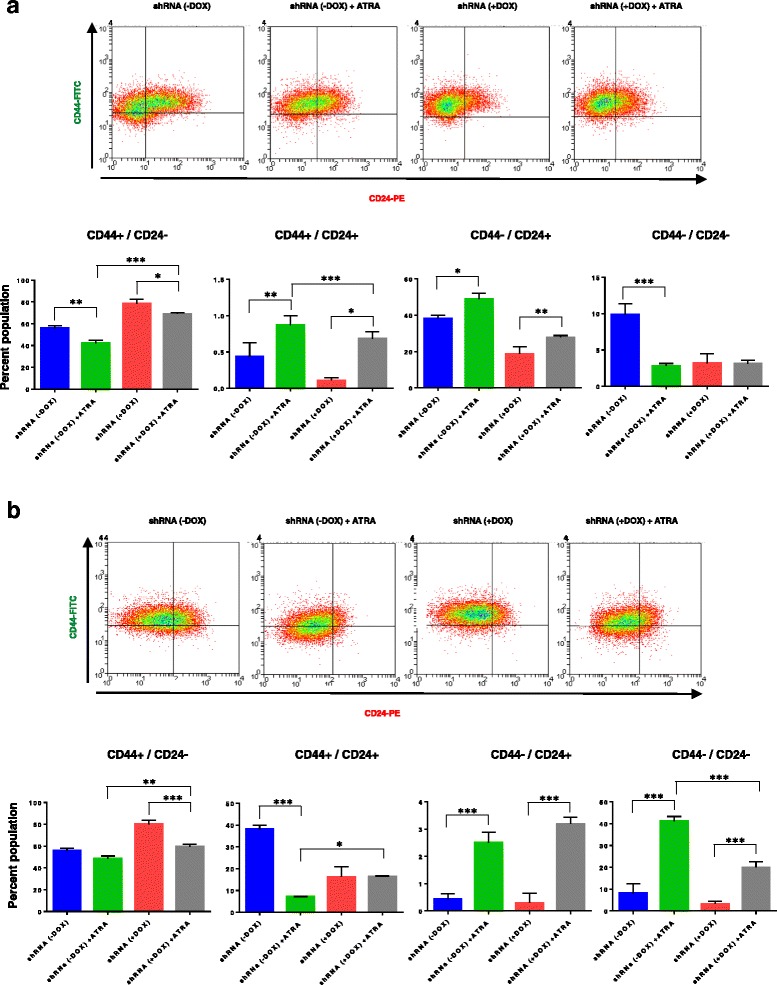
HOXC8 downregulation prevents differentiation of MCF10A cells induced by retinoic acid. **a** Representative FACS profiles and percentage of CD44^+^/CD24^low/-^, CD44^+^/CD24^+^, CD44^−^/CD24^+^ cells after retinoic acid (ATRA) treatment for 4 days and HOXC8 knockdown induced by doxycyclin (*n* = 3). Statistical analysis was performed by One-way ANOVA followed by Bonferroni’s multiple comparisons test. *P < 0.05, **P < 0.01, ***P < 0.001. **b** Representative FACS profiles and percentage of CD44^+^/CD24^low/-^, CD44^+^/CD24^+^, CD44^−^/CD24^+^ cells after retinoic acid (ATRA) treatment for 7 days and HOXC8 knockdown induced by doxycyclin (*n* = 3). Statistical analysis was performed by One-way ANOVA followed by Bonferroni’s multiple comparisons test. *P < 0.05, **P < 0.01, ***P < 0.001

Retinoic acid signalling and HOX genes are important regulators of embryo development and tissue differentiation [5, 37] Because our findings revealed a role of HOXC8 in the regulation of breast CSC self-renewal and differentiation, we next evaluated whether HOXC8 could be involved in the differentiation of mammary cells induced by retinoic acid. For this experiment, the MCF10A-Tet-HOXC8 shRNA cell line was treated with retinoic acid for 7 days in presence or absence of doxycyclin to induce HOXC8 knockdown. As previously indicated [38, 39], *HOXC8* expression can be regulated by retinoids and therefore treatment of mammary cells with retinoic acid induced *HOXC8* expression with concomitant increase in the proportion of CD24^+^ cells after 4 days treatment or CD44^−^/CD24^−^ differentiated cells after 7 days (Fig. 8, Additional file 7: Figure S6). Importantly, HOXC8 knockdown induced by doxycycline in these cells reduced cell differentiation induced by retinoic acid and both CD24^+^ and CD44^−^/CD24^−^ cell populations, suggesting a role for HOXC8 in the regulation of stem cell fate during differentiation by retinoic acid signalling in the mammary gland.

## Discussion

The importance of developmental genes in the regulation of cancer stem cell self-renewal and plasticity is well established [1, 40]. This study investigated for the first time the expression of a panel of developmental regulatorsof the homeobox family in breast cancer stem/progenitor cells. We found that homeobox genes are deregulated at an early stage of stem cell transformation as well in different cancer molecular subtypes. We found that the majority of homeobox genes analysed were differentially expressed in transformed/non tumorigenic breast stem cells, with the majority being downregulated compared to normal MaSC. Epigenetic deregulation of homeobox genes is a frequent and early event in breast cancer. DNA methylation of the *HOX* cluster has been reported to be associated with mammary epithelial cell immortalisation, suggesting that modulation of early developmental genes plays a role in escaping cell senescence [19]. In addition, alterations in DNA methylation of several homeobox genes is found in ductal carcinoma in situ (DCIS), and low-grade breast tumours [18] [41], with those found in DCIS being associated with disease progression [42]. These findings suggest a model whereby stem cells could be the cell of origin of breast tumours, as developmental genes that are normally reversibly silenced by polycomb complexes in these cells [43] become permanently silenced via DNA methylation during carcinogenesis [40, 44, 45]. Our data support this hypothesis as the majority of downregulated homeobox genes in immortal stem cells were also found not to be expressed or expressed a low level in TN breast cancer, a molecular subtype particularly enriched in CSC. A number of homeobox genes were differentially expressed only in specific breast molecular subtypes. Particularly important is the finding that the highest number of unique differentially expressed homeobox genes were found in TN CSC as this molecular subtype of breast cancer is still poorly understood and does not respond to current conventional or targeted therapies [46, 47]. DNA methylation profile of different molecular subtypes identified high enrichment of homebox genes being hypermethylated specifically in ER^−^ which were associated with high rate of tumour recurrence and therefore likely contributing to poor patient outcome [48]. Expression of homeobox genes have been also found to be specific of TN breast cancer subtypes, with the MSL (mesenchymal-stem-like) subtype being particularly enriched [49]. Therefore these newly identified CSC homeobox genes could represent novel biomarkers that could be used for patient stratification in precision medicine and warrant further investigation.

Among the differentially expressed genes identified in this study, *HOXC8* was found consistently downregulated in CSC and therefore represented a novel putative tumour suppressor candidate. *HOXC8* was especially downregulated in TN and HER2-E CSC and it was also found to be downregulated in clinical samples of TCGA and METABRIC datasets, strengthening its significance as a potential biomarker. In line with previous studies reporting the importance of epigenetic regulation of homeobox genes during development and carcinogenesis [17], we also found that *HOXC8* silencing is associated with the expression of *miR196* and DNA methylation at the gene promoter region in CSC. Regulation of *HOXC8* via miRNA 196 has been reported in breast cancer whereby the ration of *miR196* and *HOXC8* correlates with cell migration and metastasis [36] and methylation of the *HOXC8* promoter can induce gene silencing by inducing polymerase II stalling during transcription [50].

Our results demonstrate that HOXC8 is an important regulator of stem cell self-renewal and that HOXC8 can act as modulator of cell differentiation in the breast. Using a combination of strategies and cell culture models, we show that silencing of *HOXC8* confers mammary cells with stemness potential and this can be reversed by its re-expression with induction of differentiation. The enhanced CSC population induced by downregulation of HOXC8 in mammary cells is also associated with an increase in transformed phenotype and this observation supports the notion that breast cell tumorigenicity is enhanced through acquisition of an undifferentiated state [51]. HOX proteins are transcription factors that regulate cell proliferation and differentiation during embryonic development. In normal adult tissues, they maintain tissue homeostasis by defining cell fate and lineage commitment [4, 16, 17]. Several cancers demonstrate altered expression of HOX genes but their role in transformation of stem cells and establishment of CSC is not well-known. Recently, a role in CSC regulation has been strongly established for HOXA5 in breast and colorectal cancer, whereby HOXA5 can maintain homeostasis by suppressing stemness in these tissues [52, 53].

During development, *HOXC8* is expressed in the neural tube and somatic mesoderm and its expression is essential for skeletal development [54, 55]. *HOXC8* is also expressed in the normal mammary gland [8, 56, 57] and it participates in the initiation of mammary morphogenesis [58]. Previous studies have reported an increased expression of HOXC8 during breast cancer progression that can induce metastasis through direct regulation of CDH11 and EMB genes [25, 59]. This different role of HOXC8 suggests that it is possible that this gene could act as a “modulator” of carcinogenesis and therefore play either a role as suppressor or driver of transformation depending on the physiological and pathological context. Consistent with this, HOXC8 has been implicated in different cancer types, including prostate, cervical, breast, oesophageal, and pancreatic cancer [36, 60–63]. In these cancers *HOXC8* has been reported to act either as tumour suppressor gene or oncogene, suggesting that the function of *HOXC8* may depend on the activation or repression of different HOXC8 targets whose expression can be tissue-specific. Several targets of HOXC8 have been characterised [57, 64] and their functional role in the context of HOXC8 regulation may provide new insights into the role of HOXC8 in different cancer types. Although an effective approach for inhibition of the interaction between HOX proteins and co-factors PBX is available [65], targeting specific HOX proteins still remains a challenge. Therefore, a better definition of HOX transcriptional regulators and HOX targets could offer new strategies for therapeutic intervention.

We have also shown that HOXC8 downregulation in mammary cells can lead to impaired differentiation in response to retinoic acid, and that the expression of *HOXC8* can be induced by retinoids in normal breast cells. This role has also been recently demonstrated for *HOXA5*, suggesting a prominent role of HOX genes in the differentiation of the mammary gland [52]. Retinoic acid has been shown to regulate breast CSC differentiation, reduce mammospheres formation and CSC frequency in combination with chemotherapy [66, 67]. Therefore our study adds to the evidence that HOX genes are important regulators of stem cell fate and that differentiation therapy should be considered as a viable approach to target breast CSC.

## Conclusions

This study reports the screening of a large number of homeobox genes in breast CSC. We show that the expression of homeobox genes is altered in early-transformed breast cells and in different breast cancer molecular subtypes. Altered expression of homeobox genes was also supported by data obtained from large cohorts of patient through bioinformatics analysis. Therefore, this screening provides novel biomarkers that can be used in precision medicine and as possible targets for CSC-directed therapies. Of the altered genes, *HOXC8* demonstrated to function as a possible novel tumour suppressor in breast CSC by regulating stem cell self-renewal, differentiation and transformation. Therefore, this study demonstrates the critical role of homeobox genes in stem cells and paves the way to larger functional studies to explore the networks involved in homeotic gene regulation in breast cancer.

## Additional files

Additional file 1: Table S1. Primers and assays used in the study. (DOC 41 kb)
Additional file 2: Figure S1. Number of upregulated and downregulated homeobox genes in immortalised stem cells and cancer stem cells. a Proportion of up-and downregulated genes in immortalised mammary stem cells (IMM) and CSC of different breast cancer molecular subtypes as determined by Homeobox gene expression array. b Number of downregulated and upregulated genes which are unique or common to CSC of breast cancer molecular subtypes. c Venn diagrams representing downregulated and upregulated genes in stem cells of different breast cancer molecular subtypes. (PDF 204 kb)
Additional file 3: Figure S2. Data heatmap and histograms showing expression of homeobox genes in the PAM50 (TCGA WGA) (a,b,c,d) and METABRIC datasets (e,f,g,h). Yellow and blue colour intensity in the genomic heatmap indicate high and low expression, respectively. The red and green colours under the genomic heatmap indicate the statistics track showing the logarithmic plot of *P*-values for each gene using Student’ *t*-test followed by Bonferroni’s correction. Bars above the line indicates that the red subgroup (Tumour sample) is greater than the green subgroup; a bar below the line indicates that the green subgroup (Normal sample) is greater than the red subgroup. Bars are coloured in red or green when *P* < 0.05. (PDF 63 kb)
Additional file 4: Figure S3.
*HOXC8* copy number variation in the breast TCGA and METABRIC studies. Bar plots and scatter plots representing the copy number variation and correlation with gene expression in patient samples from the TCGA and METABRIC datasets [29, 30, 33, 34]. (PDF 271 kb)
Additional file 5: Figure S4. Sequencing chromatograms of bisulfite converted *HOXC8* CpG island PCR products. Bisulfite converted DNA extracted from CSC was amplified by PCR and then directly sequenced. Chromatograms show the average DNA methylation of the PCR products. CG residues are circled. A TG peak indicated unmethylated C, T/G peak indicates partially methylated C, CG peak indicates methylated C. (PDF 311 kb)
Additional file 6: Figure S5.
*HOXC8* overexpression in TN and HER2-E cells. Overexpression of *HOXC8* was induced by lentiviral transduction with pSIN-HOXC8 vector and gene expression measured by TaqMan® qRT-PCR. Results are presented as relative fold expression relative to *RPLP0* and control (pSIN empty) vector used as calibrator (*n* = 3). Relative fold expression levels were analysed by Unpaired Student *t*-test. ****P* < 0.001. Bottom panel represents Western Blotting of nuclear lysates from cells transduced with pSIN-HOXC8 overexpressing vector or control vector was conducted to detect the expression of HOXC8 (34 kDa) and LAMIN A/C (41-50 kDa) as loading control. (PDF 92 kb)
Additional file 7: Figure S6. Expression of *HOXC8* and *CD24* in ATRA treated cells. Expression of *HOXC8* and *CD24* in MCF10A-Tet-HOXC8 shRNA cells measured by TaqMan® qRT-PCR. Results are presented as relative fold expression relative to *RPLP0* and control (shRNA with no doxycyclin) used as calibrator (*n* = 3). Relative fold expression levels were analysed by One-way ANOVA followed by Bonferroni’s multiple comparisons test. **P* < 0.05, ***P* < 0.01, ****P* < 0.001. (PDF 52 kb)
