# Supplementary material for: HOXC8 regulates self-renewal, differentiation and transformation of breast cancer stem cells
Source: Mol Cancer. 2017 Feb 16;16:38. doi: 10.1186/s12943-017-0605-z (PMC5312582; doi:10.1186/s12943-017-0605-z)
Supplement: Additional file 1: Table S1. — Primers and assays used in the study. (DOC 41 kb) [file 12943_2017_605_MOESM1_ESM.doc]

**Table S1: Real time-PCR assays and primers**

|  | **Assay ID** | **Forward** | **Reverse** |
| --- | --- | --- | --- |
| **TaqMan assay** |  |  |  |
|  |  |  |  |
| *HOXC8* | Hs00224073_m1 |  |  |
| *CD24* | Hs12379687_s1 |  |  |
| *ALDH1A1* | Hs00946916_m1 |  |  |
| *ALDH1A3* | Hs00167476_m1 |  |  |
| *RPLP0* | Hs00420895_gH |  |  |
|  |  |  |  |
| **RT-qPCR** |  |  |  |
| *HOXC8 genomic* |  | GGCCATAGAGATTAGGGGTTC | GGGGCTCATGACCTAAGCTA |
| *miR196-a* |  | taggtagtttcatgttgttggg |  |
| *miR196-b* |  | taggtagtttcctgttgttggg |  |
| *RNU6B* |  | CGCAAGGATGACACGCAAATTCGTG |  |
| miR universal |  | GAATCGAGCACCAGTTACGCA |  |
|  |  |  |  |
| **Bisulfite sequencing** |  |  |  |
| *HOXC8* |  | TTTTTAATTTAGGTTATTAGTAGAATT | TTTAATTTAAATAACCTTATCCTTC |
|  |  |  |  |
| **Cloning** |  |  |  |
| *HOXC8-pSIN* |  | TTTACTAGTACCATGAGCTCCTACTTCGTC | AATTGAATTCTTAGTCCTTGTTTTCTTCCT |
| *Scrambled shRNA* |  | CCGGTCAACAAGATGAAGAGCACCAACTCGAGTTGGTGCTCTTCATCTTGTTGTTTTTG | AATTCAAAAAACAACAAGATGAAGAGCACCAACTCGAGTTGGTGCTCTTCATCTTGTT |
| *HOXC8 shRNA* |  | CCGGTGCAATATCCCGACTGTAAATCCTCGAGGATTTACAGTCGGGATATTGC TTTTTG | AATTCAAAAAAGCAATATCCCGACTGTAAATC CTCGAG GATTTACAGTCGGGATATTGC |
