# Supplementary figures and images for: HOXC8 regulates self-renewal, differentiation and transformation of breast cancer stem cells
Source: Mol Cancer. 2017 Feb 16;16:38. doi: 10.1186/s12943-017-0605-z (PMC5312582; doi:10.1186/s12943-017-0605-z)

**A**

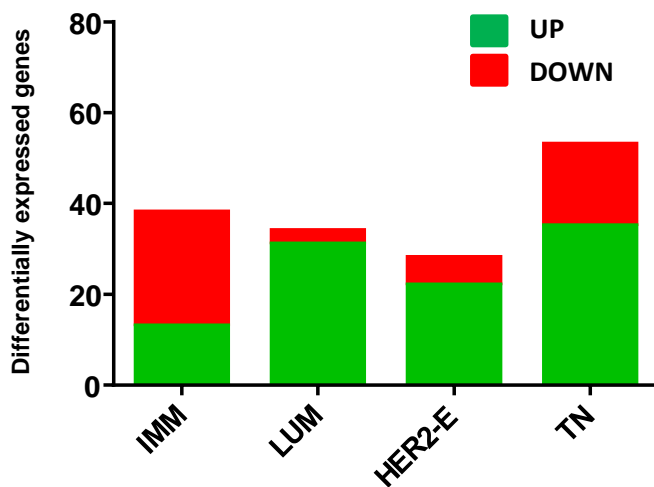

**B**

**DOWNREGULATED GENES**

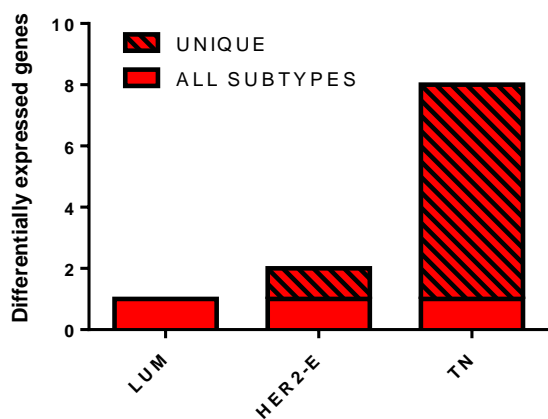

**UPREGULATED GENES**

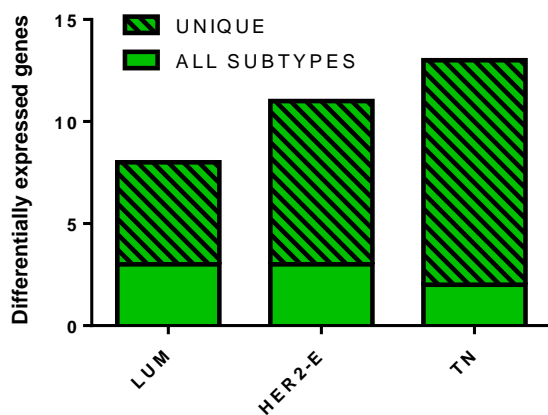

**C**

**DOWNREGULATED GENES**

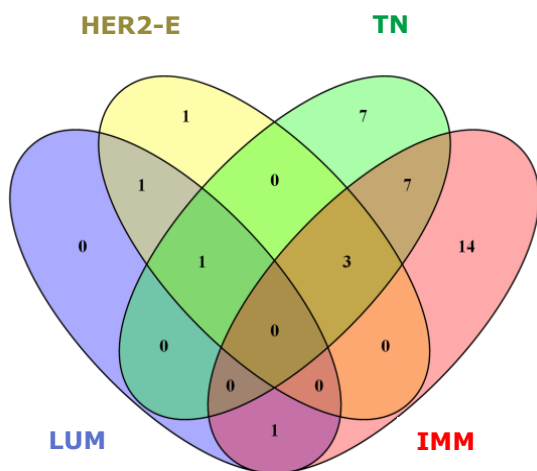

**UPREGULATED GENES**

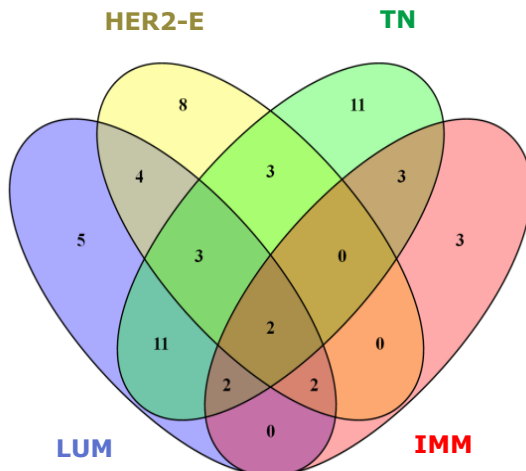

Supplement: Additional file 2: Figure S1. — Number of upregulated and downregulated homeobox genes in immortalised stem cells and cancer stem cells. a Proportion of up-and downregulated genes in immortalised mammary stem cells (IMM) and CSC of different breast cancer molecular subtypes as determined by Homeobox gene expression array. b Number of downregulated and upregulated genes which are unique or common to CSC of breast cancer molecular subtypes. c Venn diagrams representing downregulated and upregulated genes in stem cells of different breast cancer molecular subtypes. (PDF 204 kb) [file 12943_2017_605_MOESM2_ESM.pdf]

**A**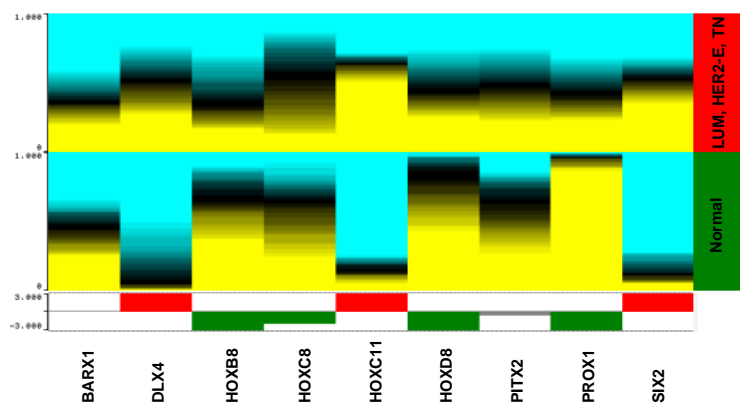**B**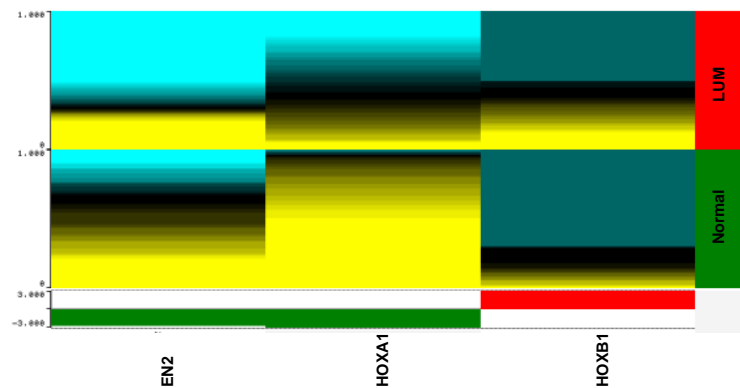**C**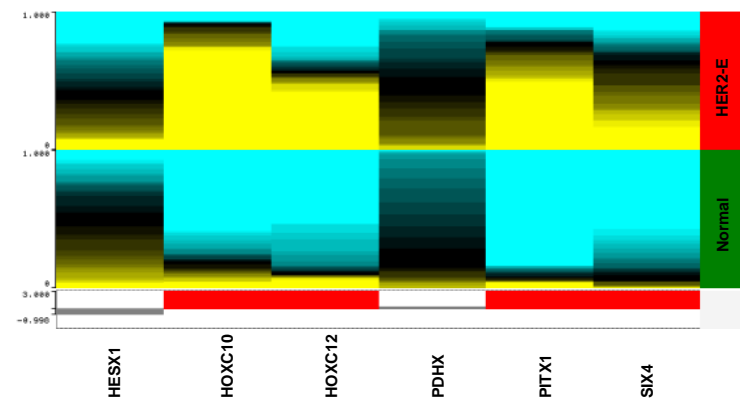**D**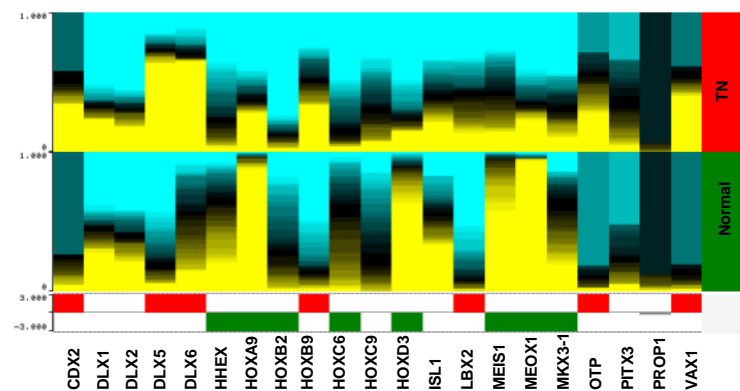**E**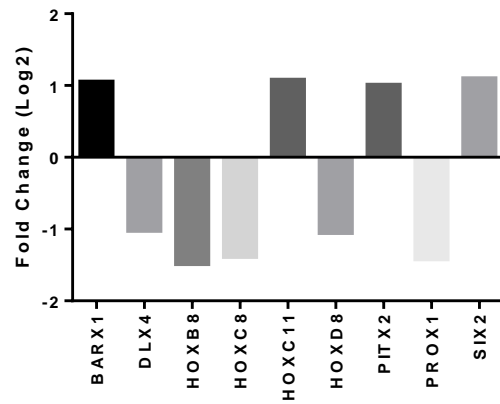**F**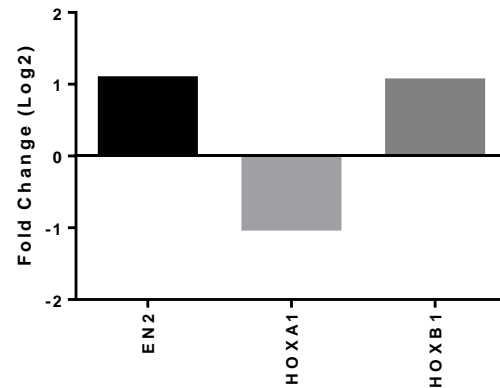**G**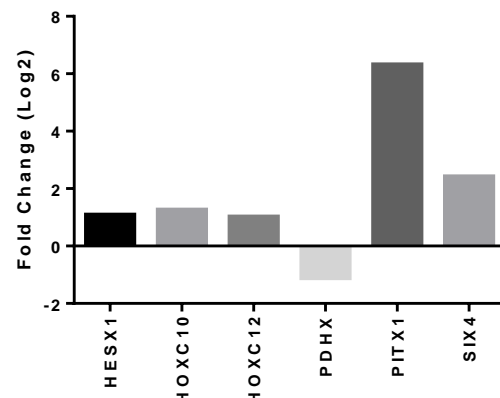**H**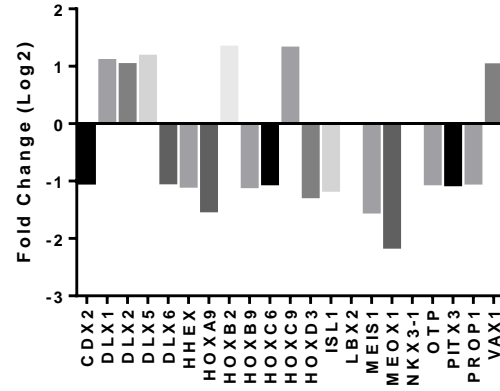

Supplement: Additional file 3: Figure S2. — Data heatmap and histograms showing expression of homeobox genes in the PAM50 (TCGA WGA) (a,b,c,d) and METABRIC datasets (e,f,g,h). Yellow and blue colour intensity in the genomic heatmap indicate high and low expression, respectively. The red and green colours under the genomic heatmap indicate the statistics track showing the logarithmic plot of P-values for each gene using Student’ t-test followed by Bonferroni’s correction. Bars above the line indicates that the red subgroup (Tumour sample) is greater than the green subgroup; a bar below the line indicates that the green subgroup (Normal sample) is greater than the red subgroup. Bars are coloured in red or green when P < 0.05. (PDF 63 kb) [file 12943_2017_605_MOESM3_ESM.pdf]

## HOXC8 Breast Invasive Carcinoma (TCGA)

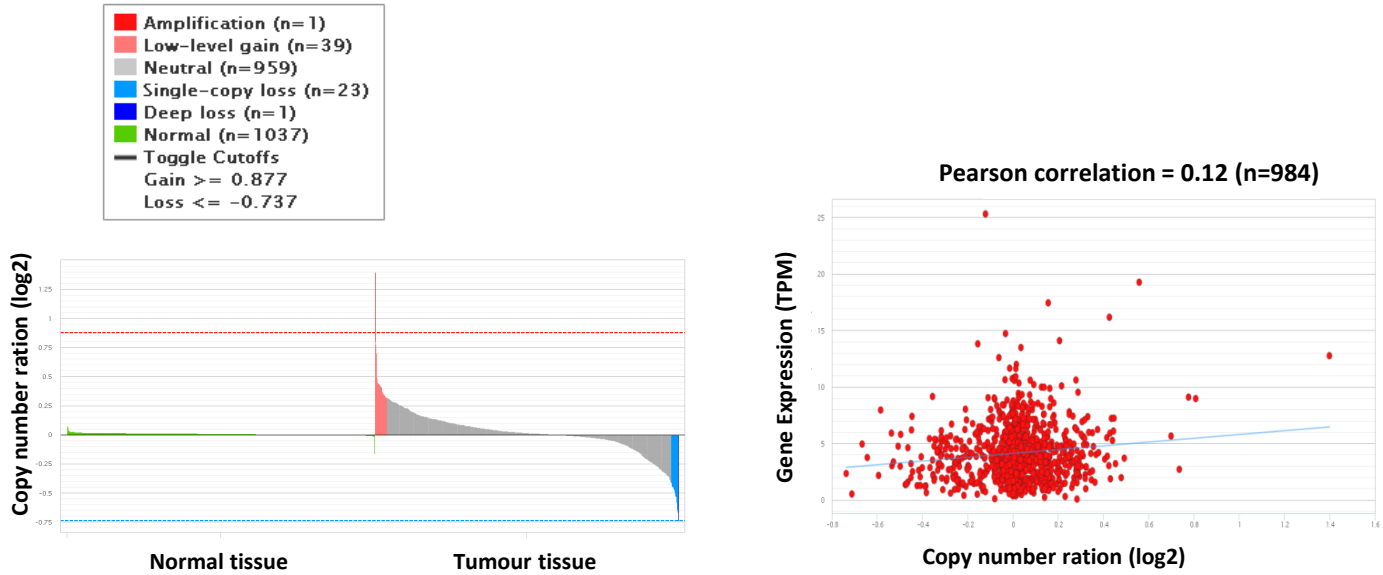

## HOXC8 Breast Cancer (METABRIC)

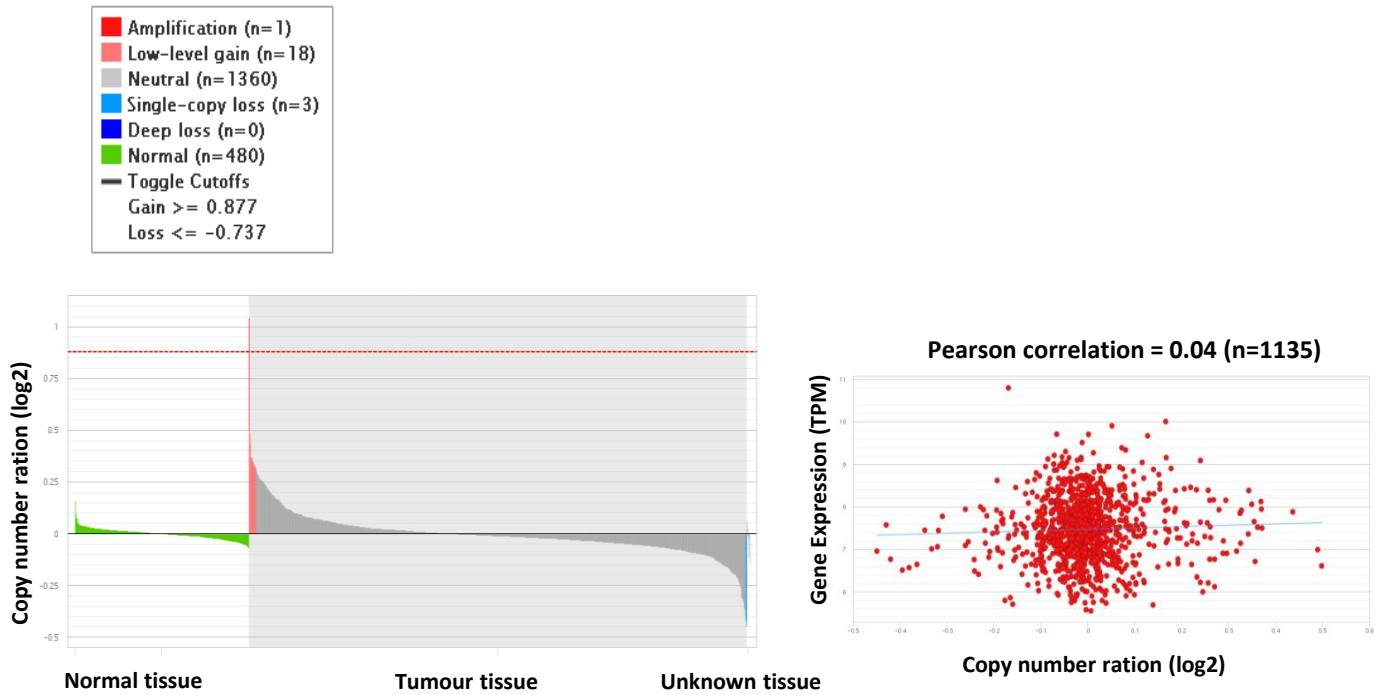

Supplement: Additional file 4: Figure S3. — HOXC8 copy number variation in the breast TCGA and METABRIC studies. Bar plots and scatter plots representing the copy number variation and correlation with gene expression in patient samples from the TCGA and METABRIC datasets [29, 30, 33, 34]. (PDF 271 kb) [file 12943_2017_605_MOESM4_ESM.pdf]

HMEC

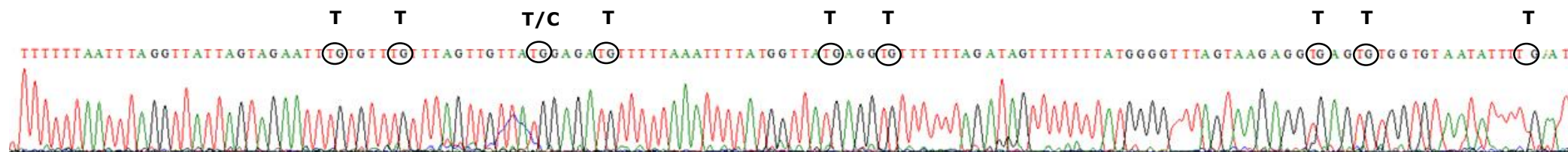

BT549

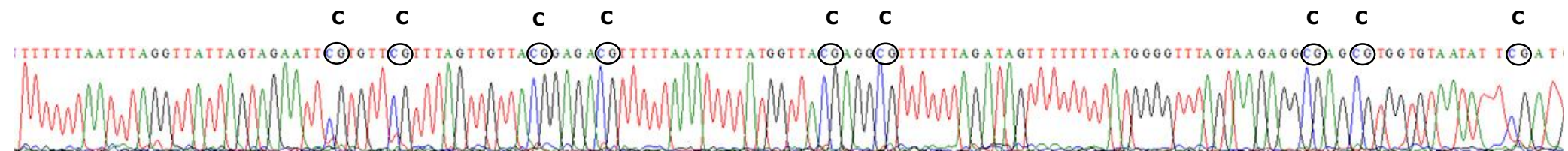

MDA-MB-231

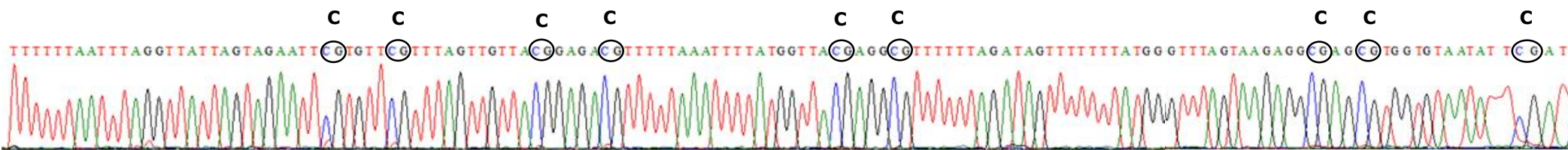

MDA-MB-468

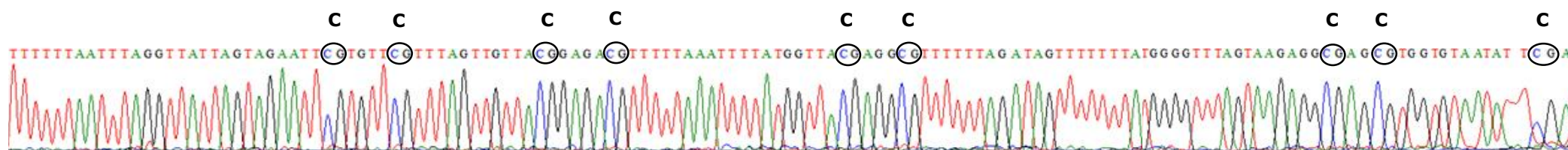

Supplement: Additional file 5: Figure S4. — Sequencing chromatograms of bisulfite converted HOXC8 CpG island PCR products. Bisulfite converted DNA extracted from CSC was amplified by PCR and then directly sequenced. Chromatograms show the average DNA methylation of the PCR products. CG residues are circled. A TG peak indicated unmethylated C, T/G peak indicates partially methylated C, CG peak indicates methylated C. (PDF 311 kb) [file 12943_2017_605_MOESM5_ESM.pdf]

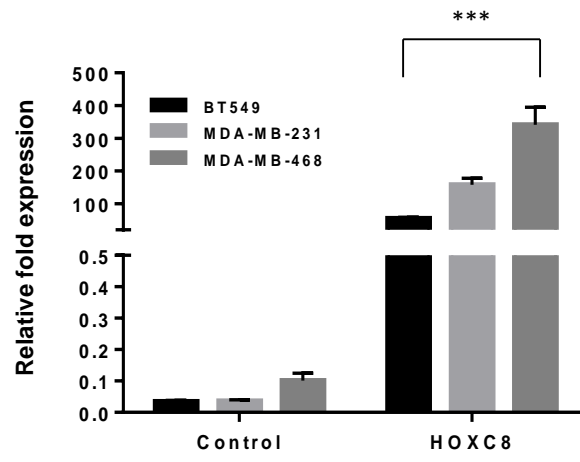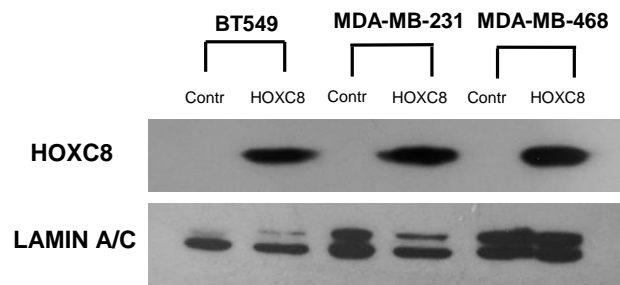

Supplement: Additional file 6: Figure S5. — HOXC8 overexpression in TN and HER2-E cells. Overexpression of HOXC8 was induced by lentiviral transduction with pSIN-HOXC8 vector and gene expression measured by TaqMan® qRT-PCR. Results are presented as relative fold expression relative to RPLP0 and control (pSIN empty) vector used as calibrator (n = 3). Relative fold expression levels were analysed by Unpaired Student t-test. ***P < 0.001. Bottom panel represents Western Blotting of nuclear lysates from cells transduced with pSIN-HOXC8 overexpressing vector or control vector was conducted to detect the expression of HOXC8 (34 kDa) and LAMIN A/C (41-50 kDa) as loading control. (PDF 92 kb) [file 12943_2017_605_MOESM6_ESM.pdf]

**HOXC8-day 4**

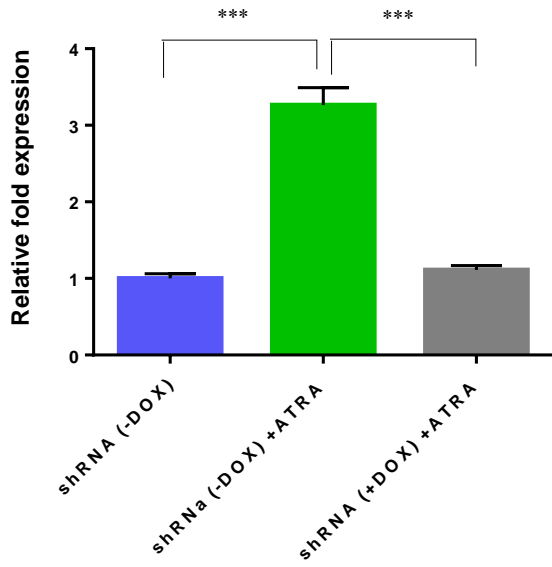

**HOXC8-day 7**

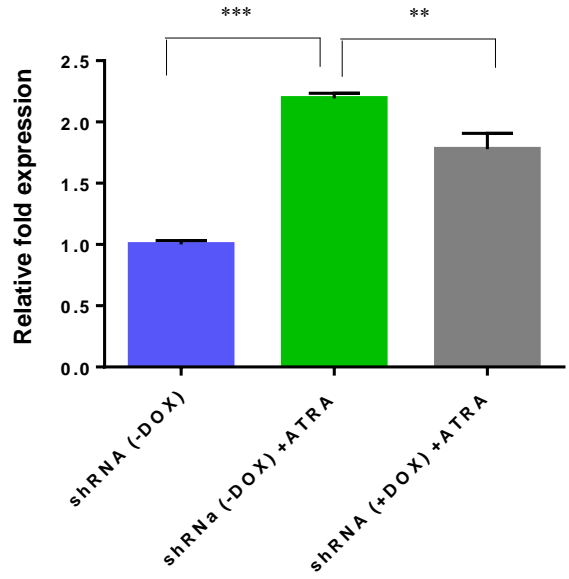

**CD24-day 4**

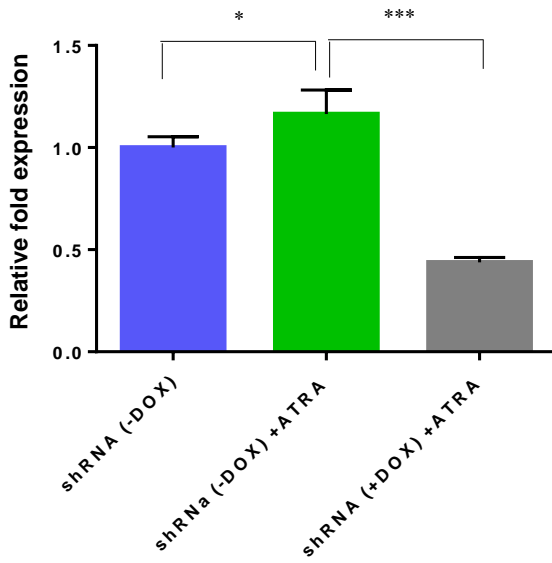

**CD24-day 7**

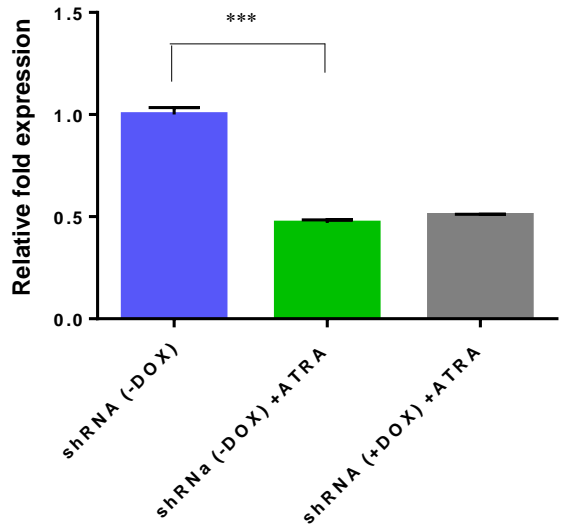

Supplement: Additional file 7: Figure S6. — Expression of HOXC8 and CD24 in ATRA treated cells. Expression of HOXC8 and CD24 in MCF10A-Tet-HOXC8 shRNA cells measured by TaqMan® qRT-PCR. Results are presented as relative fold expression relative to RPLP0 and control (shRNA with no doxycyclin) used as calibrator (n = 3). Relative fold expression levels were analysed by One-way ANOVA followed by Bonferroni’s multiple comparisons test. *P < 0.05, **P < 0.01, ***P < 0.001. (PDF 52 kb) [file 12943_2017_605_MOESM7_ESM.pdf]
